# Influence of geometry, reinforcement, and sterilisation on the dimensional accuracy of additively manufactured carbon fibre-reinforced nylon composites

**DOI:** 10.1038/s41598-025-16696-w

**Published:** 2025-10-01

**Authors:** Giles Michael Cheers, A. Goodwin, A. Heede, J. Milite, M. Johnston, A. Morris, M.-L. Wille, J. P. Little, Sinduja Suresh

**Affiliations:** 1 Department of Orthopaedics and Trauma Surgery, Musculoskeletal University Center Munich (MUM), LMU University Hospital, Munich, Germany; 2 Centre for Biomedical Technologies, Queensland University of Technology, Brisbane, Australia; 3 ARC ITTC for Multiscale 3D Imaging, Modelling and Manufacturing (M3D Innovation), Queensland University of Technology, Brisbane, Australia; 4 School of Mechanical, Medical, and Process Engineering, Faculty of Engineering, Queensland University of Technology, Brisbane, Australia; 5 Stryker Trauma GmbH, Schoenkirchen, Germany; 6 Additive Manufacturing Facility, Queensland University of Technology, Brisbane, Australia; 7 Design and Fabrication Research Facility, Queensland University of Technology, Brisbane, QLD Australia; 8 Biomechanics and Spine Research Group (BSRG), Queensland University of Technology, Brisbane, Australia

**Keywords:** Additive manufacturing, Continuous fibre reinforced polymers, Dimensional accuracy, Quality management systems, Medical devices, Biomedical engineering, Implants, Implants

## Abstract

Dimensional accuracy is a critical quality metric in manufacturing, particularly for medical devices subjected to sterilisation and disinfection. While additive manufacturing (AM), especially fused filament fabrication (FFF), facilitates the production of complex geometries, challenges such as void formation, surface deformation, and mechanical instability persist. This study evaluated the impact of sterilisation (autoclaving) and disinfection (ethanol) on the dimensional stability of 3D-printed carbon fibre-reinforced polymer (CFRP) parts. Two geometries – representing standard ASTM D3039 and complex non-standard designs – were printed using carbon fibre nylon-based composites with and without continuous carbon fibre (CCF) reinforcement. Dimensional accuracy and void fraction were assessed using micro-CT imaging and geometrical comparison analysis. While sterilisation (*p* = 0.247) and disinfection treatments (*p* > 0.05) had negligible overall effects on dimensional stability and void fraction, geometric design (*p* = 0.0036) and CCF inclusion (*p* = 0.0042) significantly influenced shape fidelity. The inclusion of CCF reinforcement enhanced resistance to deformation under external stressors, though its efficacy varied with design complexity. A significant interaction between geometry and CCF inclusion (*p* < 0.0001) demonstrated the dependency of void formation on design complexity and reinforcement. Additionally, maximum surface deviation was independently influenced by geometry (*p* = 0.0139) and CCF reinforcement (*p* = 1.1 × 10⁻⁴). This study highlights the strategic imperative of design optimisation and informed material selection to increase precision in additive manufacturing. By addressing the confluence of manufacturing constraints and stringent regulatory mandates, this research reinforces the viability of additive manufacturing for medical device fabrication, advocating for customised methodologies to harmonise functionality with compliance requirements.

**Supplementary Information:**

The online version contains supplementary material available at 10.1038/s41598-025-16696-w.

## Introduction

Fused filament fabrication (FFF) has emerged as one of the most democratised routes to three-dimensional (3D) printing, pairing low capital investment with the capacity to translate highly intricate computer-aided design (CAD) intent into functional objects^1^. By extruding thermoplastic filaments in a layer-wise manner along a G-code-defined tool path, the technology offers design engineers—and increasingly clinicians—an avenue to fabricate bespoke parts on demand. The maturation of slicing algorithms, sensor-guided feedback loops, and multi-material extrusion heads has elevated FFF from its prototyping origins to a bona-fide manufacturing flatform within the broader additive manufacturing (AM) ecosystem.

In recent years, continuous fibre-reinforced polymer (CFRP) composites have been transformative materials within the aerospace and medical sectors. The integration of continuous fibres within a thermoplastic matrix during the FFF process has enabled the creation of 3D-printed CFRP components^2^providing a viable alternative to conventional metal-based materials. By combining lightweight attributes with superior mechanical properties, CFRPs achieve exceptional strength-to-weight ratios, making them ideal for applications demanding structural integrity and reduced mass^3^. The advent of AM has further accelerated CFRP adoption, enabling the rapid fabrication of geometrically complex and functional components with reduced lead times^4^.

Among the various reinforcement fibres, carbon fibre has garnered widespread attention due to its high modulus-to-density ratio, radiolucency, and favourable cytocompatibility profile^5^. These characteristics have catalysed its deployment in drill guides, retractor arms, and even load-bearing implants^2,6,7^. Yet the clinical translation of CFRP devices hinges not merely on mechanical superiority but on the material’s resilience to sterilisation and disinfection workflows—processes mandated to eradicate pathogens prior to patient contact^8^. Each protocol—thermal, chemical, or radiation-based—imparts its own thermomechanical or chemomechanical effect, potentially perturbing geometry, microstructure, or both.

Despite the growing utilisation of 3D printing in medical contexts, systematic insight into how sterilisation and disinfection influence 3D-printed CFRPs remains fragmentary. Existing literature disproportionately examines homopolymers such as PLA, ABS, and PEEK or photopolymer resins^9–16^; the multi-phase nature of CFPRs introduces additional failure modes. For instance, the disparity in coefficients of thermal expansion (CTE) between the fibres and the polymer matrix can cause differential thermal expansion, leading to dimensional instability^17^. Additionally, weak adhesion at the fibre-matrix interface, because of poor thermal conductivity and insufficient binding materials, can exacerbate interfacial instability under thermal or chemical stress^18^. Accordingly, quantifying how sterilisation and disinfection perturbs both the continuum scale (dimensional fidelity) and the mesoscale (void architecture) is essential for risk assessment in regulatory submissions^13^.

The dimensional accuracy of fabricated parts is considered a main quality control (QC) indicator in the manufacturing engineering process, referring to the degree to which a fabricated part conforms to its intended design dimensions^19^. It is particularly important for interconnecting parts to ensure a precise fit, allowing seamless assembly and ensuring desired functionality. Any deviations in dimensions can lead to misalignments, affecting the performance and integrity of the interconnected components. However, inherent challenges in FFF, such as voids, surface roughness, and weak fibre-matrix bonding, collectively reduce the material’s density and compromise its mechanical performance^20,21^. The careful selection of printing parameters tailored to the specific printer and filament type is thus vital to mitigate these shortcomings.

Given these considerations, the present study investigates how two ubiquitous, cost-conscious protocols—steam autoclave sterilisation and 70% ethanol immersion—reshape the geometry and internal porosity of 3D-printed carbon-fibre nylon composites. Autoclave sterilisation provides a robust test of material integrity by subjecting parts to high thermal and pressure-based stresses, reflecting its prominence as a standard sterilisation method in clinical practice. Conversely, ethanol submersion is particularly relevant due to its widespread use in disinfection protocols, its compatibility with certain medical devices, and its tendency to infiltrate polymer matrices, potentially causing swelling and dimensional deviations. Understanding this behaviour is critical, as chemical disinfectants are frequently used in medical settings during pre-use cleaning or reuse protocols. We deliberately contrast a benchmark ASTM-D3039-derived coupon with a bespoke, clinically inspired geometry that concentrates features notorious for inducing print-quality drift (threads, serrations, and variable wall thicknesses). Moreover, we evaluate each geometry in two material states: micro-carbon-fibre-filled nylon and its continuous-carbon-fibre (CCF) reinforced analogue. By examining the interplay between sterilisation and disinfection conditions, material composition, and geometrical structure, this work aims to elucidate the capacity of CFRPs to maintain dimensional fidelity, thereby advancing their suitability for precision-driven medical applications.

## Materials and methods

### Part design and fabrication

Two sample geometries were designed via SolidWorks (v.2022, Dassault Systèmes, Waltham, MA, USA): a standard scaled rectangular geometry (S) and a bespoke non-standard geometry (NS). The standard geometry adhered to the ASTM D3039 specifications for evaluating the tensile properties of polymer matrix composite materials and had dimensions of 40 × 16.50 × 2.90 mm. The non‑standard coupon was intentionally designed as a compact surrogate for several feature classes that recur in additively manufactured medical hardware. The outer helical swale reproduces the shallow, compound‑curvature surfaces found on contoured fixation plates; the inner ratchet‑style teeth emulate gripping or locking mechanisms in arthroscopic anchors and spinal cages; and the ring topology imposes a continuous thin wall that challenges dimensional stability during circumferential tool‑path changes. By combining fine threads, sharp serrations, overhangs and variable wall thickness in a single part small enough for the micro‑CT field of view, the geometry provides a stringent, yet clinically representative, test bed for void formation and shape‑fidelity under sterilisation and disinfection.

**Fig. 1:**
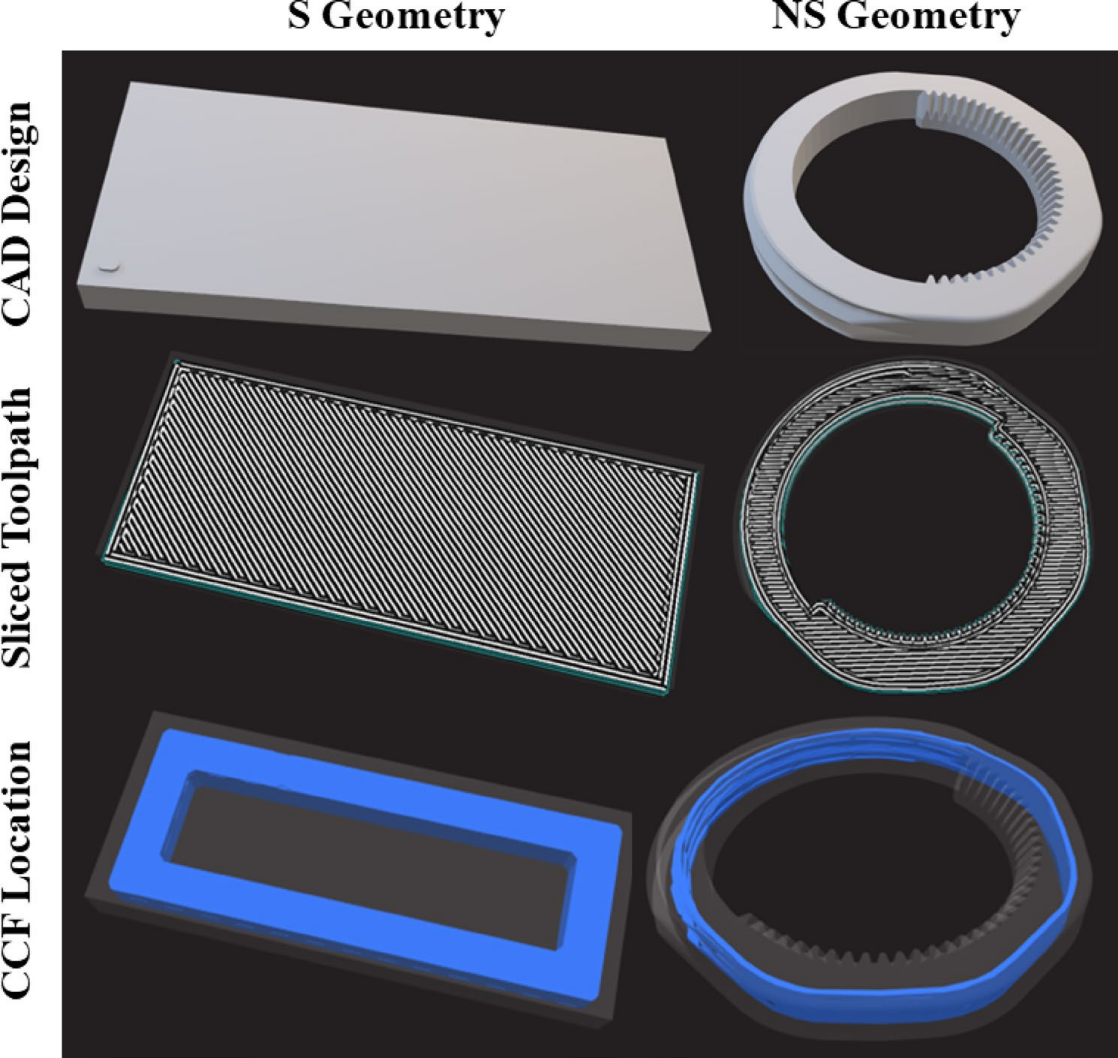
CAD models of the standard (left) and non-standard (right) geometries used in the study. Blue rings indicate the concentric CCF deposition paths.

The samples were created via FFF technology within the closed-parameter Markforged ecosystem (Markforged, Waltham, MA, USA), using two primary materials: Onyx^®^ and Onyx^®^ reinforced with CCF. Onyx^®^ is a thermoplastic polymer composite that contains micro carbon fibre-filled nylon and caprolactam in a proprietary ratio. Model preparation was executed in Eiger™ slicing software. We selected the vendor-validated solid fill profile, which applies 100% rectilinear infill with a −45°/45° raster alteration, two perimeter shells, two top and bottom layers, and a layer height of 0.15 mm. For samples containing CCF, the default number of concentric CCF rings (*n* = 2) was used (visualised in Fig. 1). The FFF nozzle is 0.40 mm in diameter, the CFF nozzle 0.90 mm; both travel under firmware‑controlled feed rates of 22–32 mm/s for this layer height.

Manufacturing was carried out via a Markforged Mark Two™ 3D-printer, which operates by extruding heated filament at 275 °C in a sequential layer-by-layer process. The non‑standard geometry required same‑material supports, generated automatically at the slicer’s 50° overhang threshold; the ASTM‑derived coupon printed without supports. In samples where CCF was utilised, a second extrusion nozzle deposited CCFs between the two walls of printed Onyx^®^.

The Markforged workflow locks most slice variables to pre‑qualified values, removing the need (and possibility) for manual tuning. Our optimisation therefore consisted of (i) selecting the vendor’s dimensional‑accuracy profile (0.15 mm layers) and (ii) choosing build orientation and support generation.

**Fig. 2:**
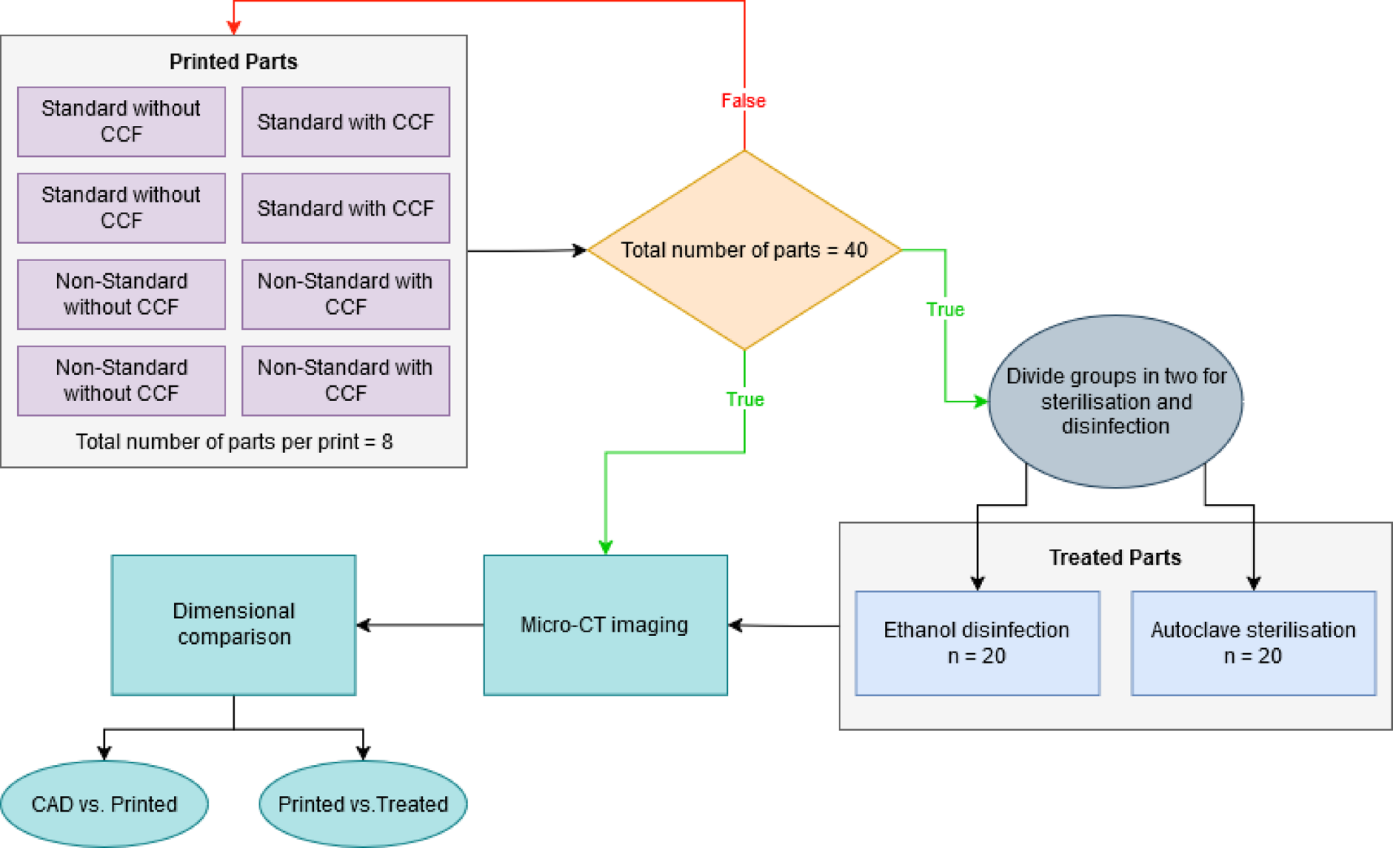
Experimental workflow for fabrication, sterilisation/disinfection, micro-CT scanning, and dimensional metrology of nylon/carbon-fibre composite parts, with and without CCF, in both geometries.

The production was divided into five distinct print jobs, each generating eight samples. Each printing task comprised the following: two standard geometry samples composed of Onyx^®^, two standard geometry samples composed of Onyx^®^ with CCF, two non-standard geometry samples composed of Onyx^®^, and two non-standard geometry samples composed of Onyx^®^ with CCF. This thorough printing programme ensured the preparation of two samples from each category for subsequent evaluation under sterilisation and disinfection methodologies, culminating in a total of forty samples. This sample size was chosen to ensure sufficient representation across all material and geometry combinations, allowing for robust evaluation of dimensional stability under sterilisation and disinfection protocols. The entire workflow for fabricating, sterilising and disinfecting, scanning and dimensional analyses is shown in Fig. 2.

### Micro-CT of the printed samples (micro-CT models)

Each sample underwent scanning using a Scanco micro-CT 50 machine (Scanco Medical AG, Switzerland) both pre- and post-treatment. The samples were placed in a Ø 48 mm x H 110 mm sample tube with low-density packing foam and were scanned at an isotropic voxel size of 24 µm^3^ with an exposure time of 0.6 s. The X-ray source voltage and beam current were set at 55 kVp and 145 µA, respectively and a 0.1 Aluminium filter was used. The data from each scan was evaluated and extracted as digital imaging and communications in medicine (DICOM) images for 3D segmentation. These images were then reconstructed and segmented using Materialise Mimics software (v.21.0, Materialise, Leuven, Belgium), employing global thresholding with a minimum Hounsfield Unit (HU) value of 850 HU. Tools such as ‘split mask’ were employed to eliminate residual noise from beam hardening prior to processing the final mask. Segmented and refined masks were converted to Binary Little Endian mesh files (STL) at a ‘medium’ mesh resolution for geometric accuracy analysis against the original CAD models.

### Sterilisation and disinfection

Each pair of printed parts were divided into ethanol disinfection and steam autoclaving groups.

Ethanol disinfection involved immersing the samples in a 70% ethanol solution maintained at 20 °C for 20 min. After immersion, the samples underwent five washes with sterile phosphate-buffered saline (PBS) on a plate shaker, each lasting five minutes. Subsequently, the samples were air-dried at ambient room temperature before undergoing micro-CT scanning. This procedure was adapted from established disinfection protocols routinely employed for medical devices before and after surgical procedures, in accordance with the guidelines outlined in ISO 17664-1:2021, which specify that medical device manufacturers should provide detailed processing information for products intended for invasive use or direct/indirect patient contact^10,11,13,22,23^.

Steam autoclaving was conducted in accordance with ISO 17665-1:2006 standards. The autoclaving process included maintaining a temperature of 121 °C at 30 psi for 30 min. Following autoclaving, the samples were air-dried at ambient room temperature prior to micro-CT analysis.

### Void fraction determination for micro-CT models

The exported DICOM datasets of the samples were reconstructed and segmented using Amira-Avizo software (v.2022.2, ThermoFisher Scientific, Waltham, MA, USA) to create 3D models of both the solid component and the void space. This process employed a threshold-based image segmentation technique with a minimum radio density set at 850 HU. The “fill holes” operation was performed across all slices to produce a representative model of the void space. The solid part of the sample was generated using the same segmentation process, ensuring the selection of the desired material.

For the non-standard geometry, post-processing involved adjusting the HU value to highlight the entire image and subtracting the circular region corresponding to the inside material. Avizo’s volume fraction tool was used to compute the volumes of each segmented part. Void fraction for each micro-CT model was calculated using Eq. (1).

1 $$\:Void\:Fraction\:\left(\%\right)=\frac{{Volume}_{void}}{{Volume}_{total}}\times\:100$$

### Geometrical comparison between CAD, manufactured, and treated samples

The quality of the printing process was assessed by evaluating the fabrication accuracy, which quantifies the congruence between the printed samples and their corresponding CAD models. This evaluation was conducted using Geomagic Control X (v.2020.1, 3D Systems Inc., USA). Micro-CT models of the printed parts before treatment were exported as STL files and imported into the software. These models were aligned with the original CAD models through an iterative alignment process. This process commenced with the initial (precise) alignment tool, followed by manual translations, and concluded with the best-fit alignment tool based on an iterative closest point (ICP) algorithm. After alignment, a geometric comparison of the meshes was performed using the ‘3D Compare’ module within the software. The comparison utilised the shortest projection direction, and an automated maximum deviation setting was applied to exclude outliers. The geometrical tolerance for the analysis was set to ± 0.15 mm, corresponding to the z-axis resolution defined by the printed layer height.

Additionally, the effect of ethanol disinfection or autoclave sterilisation on the composite polymer was assessed by comparing each pre-treatment sample with its corresponding post-treatment sample. This comparison followed the same alignment and geometric accuracy evaluation process as described above.

### Statistical analysis

The data from void fraction and geometrical accuracy analysis were descriptively statistically analysed with Microsoft Excel (v2019, Microsoft, USA). Graphs were created in BioRender. Statistical analyses were conducted to evaluate the effects of geometry, carbon fibre inclusion, and treatment method on void fraction and surface deviation in 3D-printed samples. Surface deviation (in mm) served as a measure of shape fidelity, while void fraction reflected the porosity of internal structure.

All inferential statistics were performed in MATLAB (v.R2023b, MathWorks, USA), with the threshold for statistical significance set at α = 0.05 (two-tailed). A fully factorial design was employed for statistical analysis. For both void fraction and geometric deviation, three-way ANOVA models were specified, incorporating geometry (standard vs. non-standard), carbon fibre reinforcement (present vs. absent), and sterilisation method (ethanol vs. autoclave) as fixed factors. All main effects and interaction terms, including two- and three-way interactions, were included to identify both independent and combined influences of each factor on the measured outcomes. For CAD-to-printed sample comparisons, two-way ANOVA models were utilised, limited to geometry and carbon fibre, to isolate material and design effects prior to treatment. Maximum surface deviation was similarly analysed using two-way ANOVA, restricted to geometry and carbon fibre as fixed factors, to evaluate the effect of design and reinforcement on localised surface errors. All statistical models were explicitly constructed to detect interaction effects and to interpret main effects in their presence.

Model residuals from each ANOVA were systematically assessed for normality using the Lilliefors modification of the Kolmogorov–Smirnov test, and for homogeneity of variances using Levene’s test. Where the assumption of homoscedasticity was violated, as determined by Levene’s *p* < 0.05, robustness of conclusions was validated by repeating the analysis with a non-parametric Scheirer–Ray–Hare (SRH) (rank ANOVA) test, which models main effects and interactions on ranked data. In all cases, the pattern of statistical significance was consistent between parametric and non-parametric approaches, indicating that the findings were robust to mild deviations from ANOVA assumptions.

Wherever main effects or interactions reached significance, post hoc pairwise comparisons were conducted using the Tukey Honest Significant Difference (HSD) procedure, controlling the familywise error rate. For rank-based analyses, post hoc tests were similarly performed on ranked data. Confidence intervals and exact p-values are reported for each relevant group comparison.

## Results

### In-silico void fraction assessment of the printed models

**Fig. 3:**
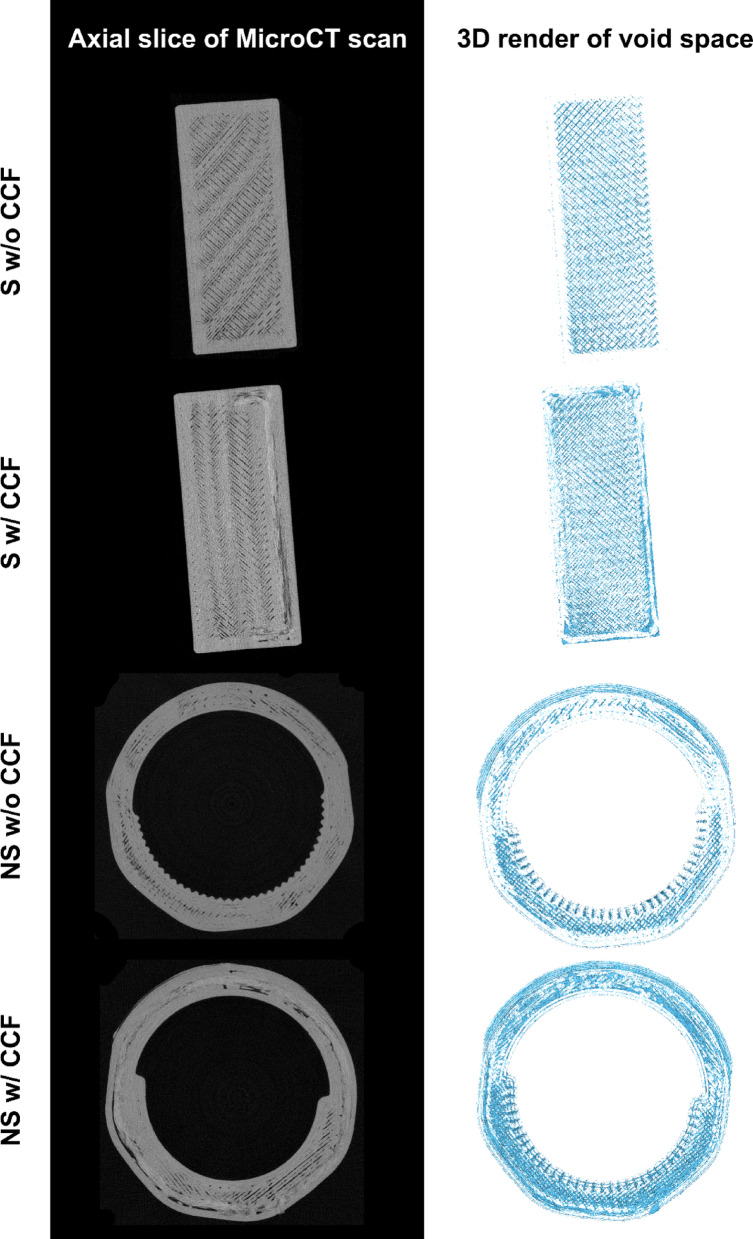
Micro-CT assessment of voiding: representative axial slices (left) and 3D reconstructions of segmented voids (right) for each material/geometry combination.

**Fig. 4:**
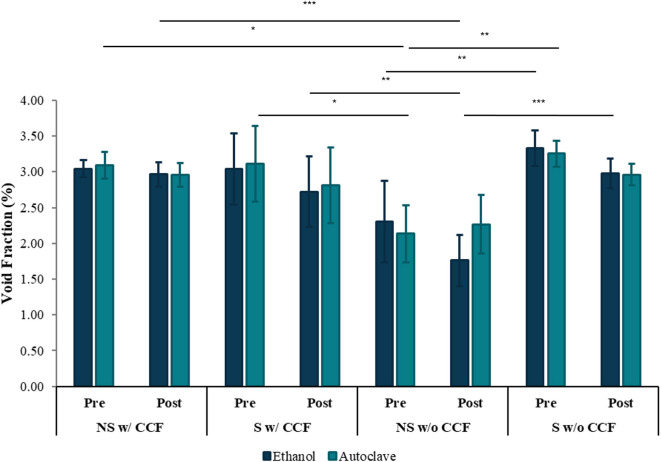
Void fraction before and after ethanol disinfection or autoclave sterilisation, stratified by geometry and CCF reinforcement. Significance markers: *p* < 0.05 (*), *p* < 0.01 (**), *p* < 0.001 (***).

Segmented samples from micro-CT imaging were used to assess the void fraction within the printed models as shown in Fig. 3. Descriptive statistics are recorded in supplementary Tables 1 and corresponding data can be visualised in Fig. 4.

Qualitative inspection of the micro-CT void maps in Fig. 3 shows a distinct, geometry‑ and reinforcement‑dependent pattern. In standard coupons without CCF, porosity manifests as uniform, gaps between rectilinear raster lines, with no localised clusters. Adding CCF introduces a second, more prominent void population: elongated cavities that trace the concentric fibre rings, while the inter‑raster gaps persist. The non‑standard coupons without CCF replicate the raster‑driven porosity but also display void densities in the threaded region and along the serrated tooth ridge. In the non‑standard coupons with CCF, voids again concentrate around the fibre rings and intensify where subsequent polymer layers bridge over these rings at sharp curvature transitions; the same areas exhibit the largest dimensional offsets. Together, these maps indicate that the observed global variability in void fraction and accuracy is rooted in repeatable, feature‑specific porosity rather than in stochastic differences between print runs.

A three-way ANOVA evaluating the influences of geometry, CCF reinforcement, and sterilisation method on void fraction revealed significant main effects for both geometry (*p* = 0.00360) and CCF inclusion (*p* = 0.00420), whereas the sterilisation method exhibited no statistically significant effect (*p* = 0.247) (supplementary Table 5). A highly significant interaction between geometry and CCF inclusion was also identified (*p* < 0.0001), demonstrating that the influence of fibre reinforcement on void formation differs according to geometric complexity. Other interactions involving sterilisation—geometry × sterilisation (*p* = 0.391), CCF inclusion × sterilisation (*p* = 0.400), and the three-way interaction (*p* = 0.210)—were not significant, indicating that sterilisation processes did not meaningfully affect void content.

Post-hoc comparisons using Tukey’s HSD test (supplementary Table 6) clarified the nature of the Geometry × CCF interaction. Specifically, the non-standard geometry printed without CCF reinforcement exhibited the lowest void fractions of all configurations, with mean values approximately 0.95% points lower than the other groups (all *p* < 0.001). In contrast, no significant differences emerged among the remaining three groups (all *p* > 0.6), indicating that either the inclusion of fibre or the use of a standard geometry elevates void formation to a similar extent.

Assessment of ANOVA assumptions confirmed equal variances across groups (Levene’s test, *p* = 0.052), though residuals deviated from normality (Lilliefors test, *p* < 0.001). To verify robustness despite this deviation, a non-parametric SRH-based test was performed and yielded an identical pattern of significance for geometry (*p* = 0.0099), fibre inclusion (*p* = 0.0099), and their interaction (*p* < 0.001), confirming the validity of the parametric results (supplementary Table 5).

### 3D geometrical comparison between the CAD model and the printed model

**Fig. 5:**
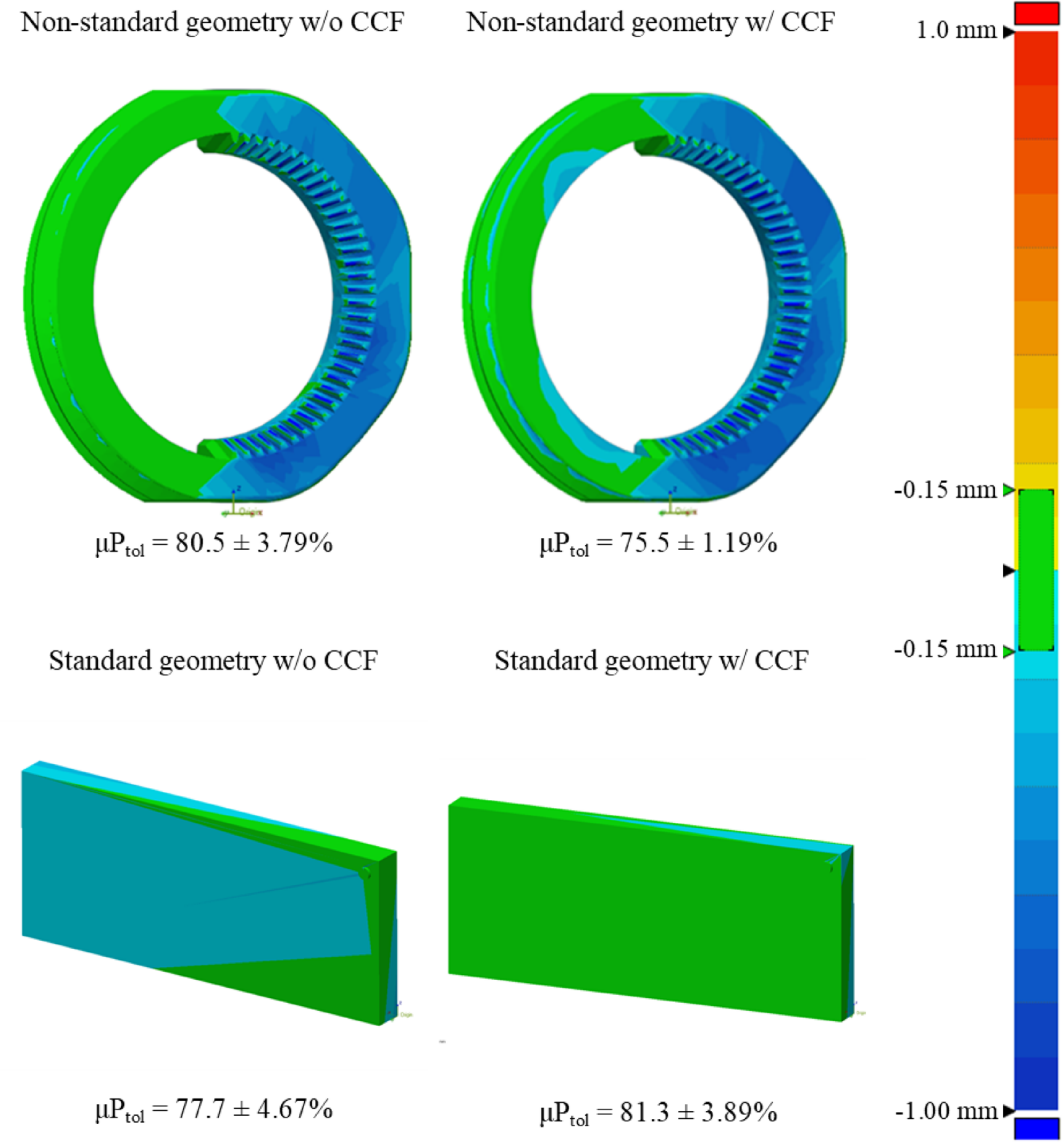
Representative surface-deviation heatmap comparing the CAD model to the as-printed part (global geometric error visualisation).

**Fig. 6:**
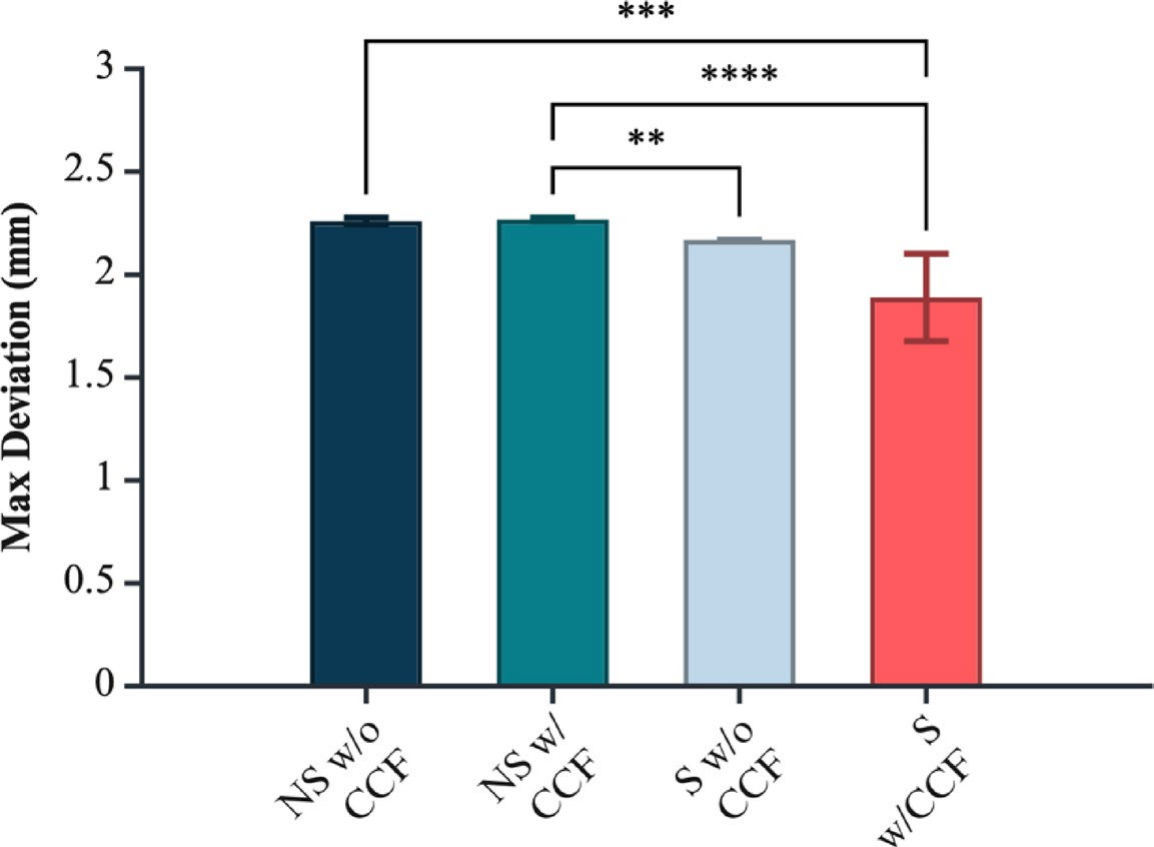
Maximum global deviation (CAD vs. pre-treatment) by geometry and CCF reinforcement. Significance markers: *p* < 0.01 (**), *p* < 0.001 (***), *p* < 0.0001 (****). Figure created with BioRender.com.

**Fig. 7:**
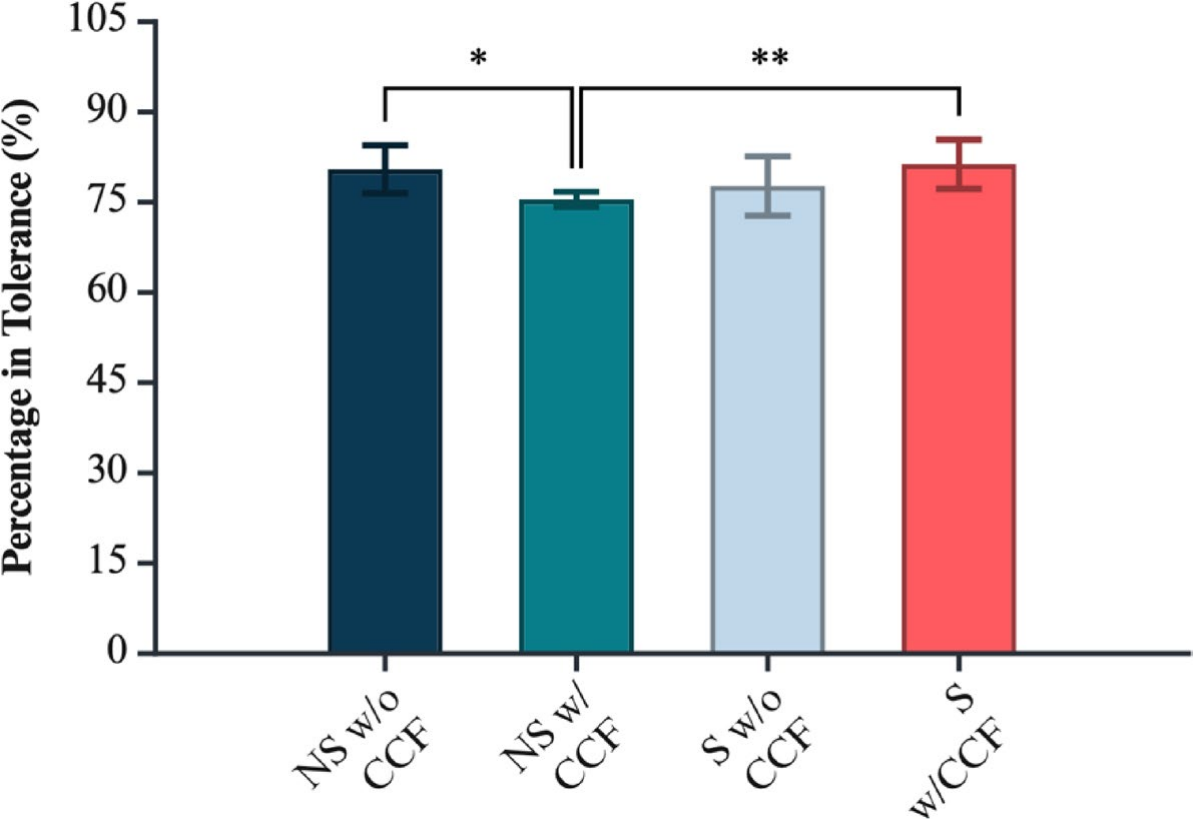
Percentage of surface area within tolerance (CAD vs. pre-treatment) for each geometry/CCF group. Significance markers: *p* < 0.05 (*), *p* < 0.01 (**). Figure created with BioRender.com.

Segmented samples from micro-CT imaging were used to assess the geometrical accuracy of printed models against the original CAD models as shown in Fig. 5. Descriptive statistics are recorded in supplementary Tables 2 and the corresponding data can be visualised in Figs. 6 and 7.

Maximum deviation from the reference CAD file was determined by both the macro-geometry of the coupon and the presence of CCF rings, with a pronounced, geometry-dependent interaction between the two factors. Non-standard coupons exhibited greater geometrical error than planar ASTM plates (*p* = 3.63 × 10⁻⁸), while CCF inclusion also significantly influenced deviation across all geometries (*p* = 2.80 × 10⁻⁴). The significant Geometry × CCF interaction (*p* = 1.49 × 10⁻⁴) demonstrates that the effect of fibre reinforcement is not uniform: its impact on geometrical fidelity depends critically on the underlying geometry.

Assumption checks showed normal residuals (Lilliefors *p* = 1.00) but unequal variances among groups (Levene *p* < 0.001). To confirm robustness, a variance-robust SRH rank test reproduced the same pattern of significance (geometry *p* = 5.66 × 10⁻¹⁶, CCF *p* = 0.042, interaction *p* = 2.72 × 10⁻⁵).

Tukey HSD (supplementary Table 6) contrasts identified the standard geometry with CCF as the group with the lowest deviation from the CAD reference, outperforming the standard geometry without CCF by 0.279 mm (*p* = 6.51 × 10⁻⁶), the non-standard geometry with CCF by 0.378 mm (*p* = 1.24 × 10⁻⁸), and the non-standard geometry without CCF by 0.371 mm (*p* = 1.93 × 10⁻⁸). All other pairwise group comparisons were non-significant (*p* > 0.179). These findings indicate that geometric complexity alone elevates error, but crucially, embedding fibre rings in a planar geometry improves surface fidelity, whereas their effect is negligible in more complex, non-standard forms.

### Regional surface comparison between the CAD model and the printed model

**Fig. 8:**
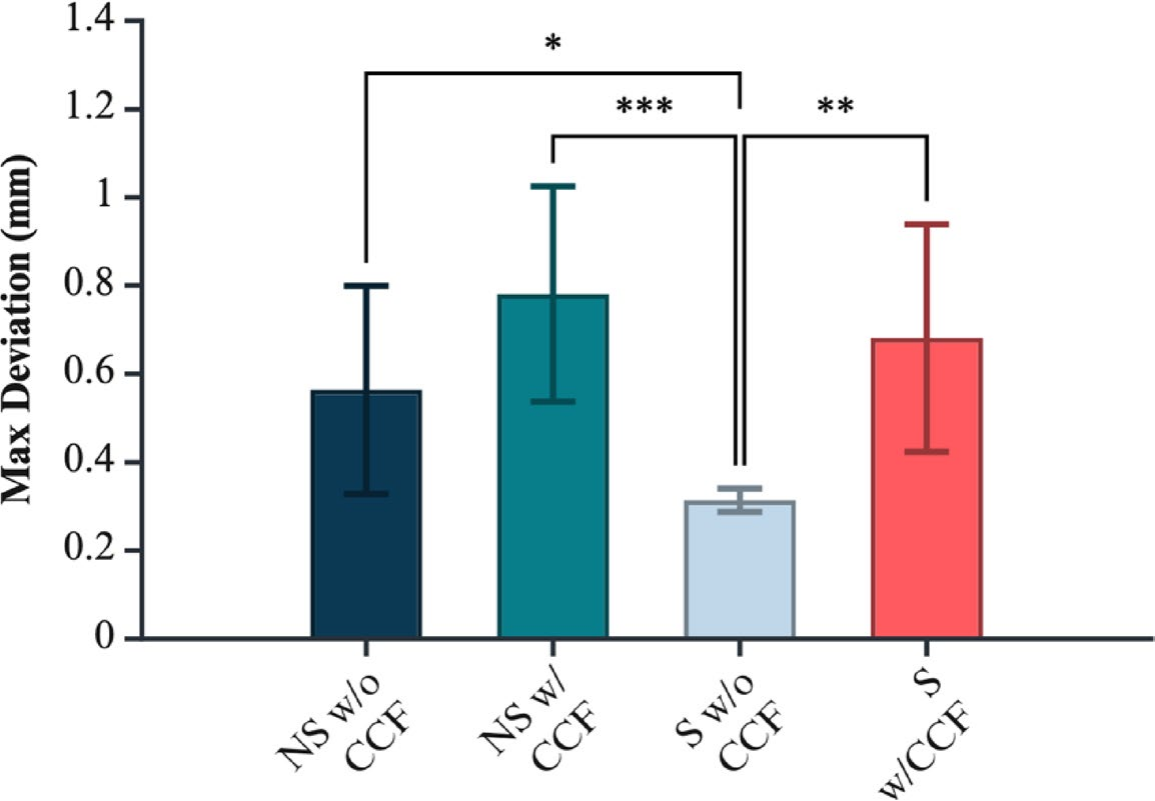
Maximum surface-only (regional) deviation (CAD vs. pre-treatment), highlighting feature-level disparities (e.g., teeth vs. threads). Significance markers: *p* < 0.05 (*), *p* < 0.01 (**), *p* < 0.001 (***). Figure created with BioRender.com.

**Fig. 9:**
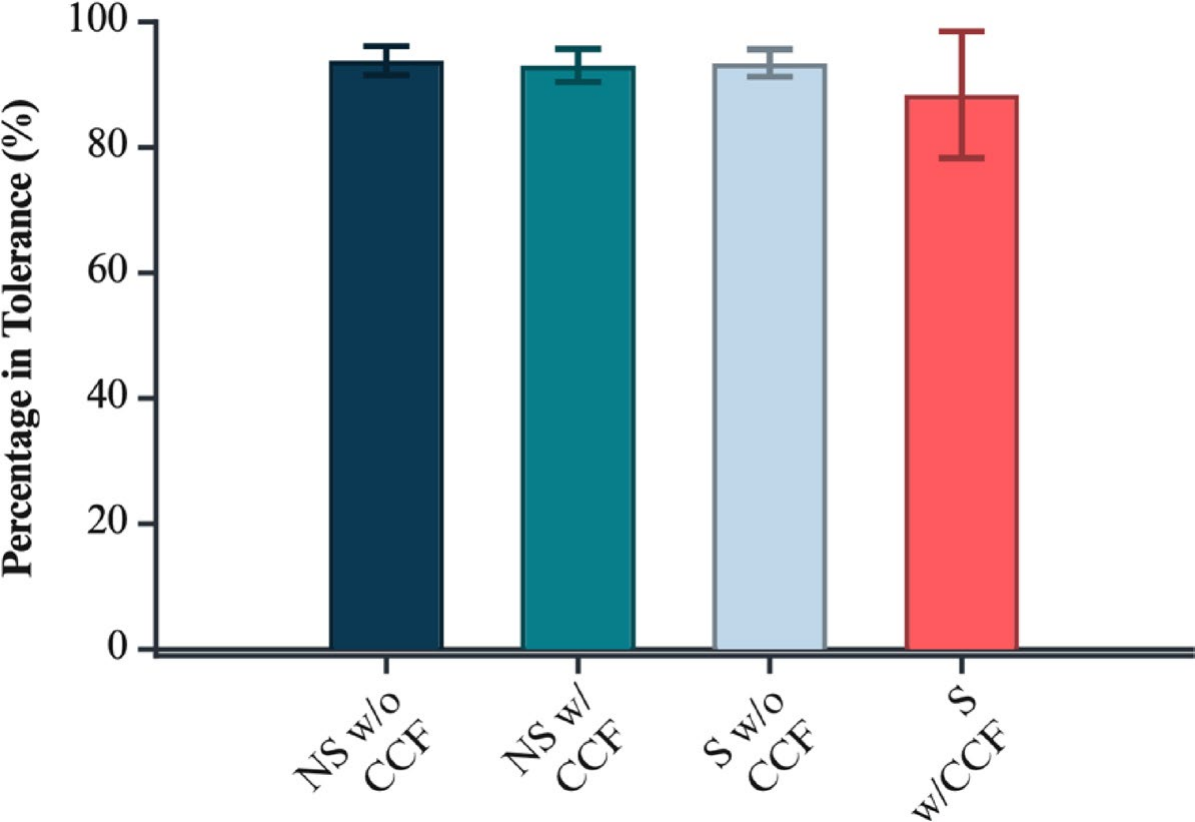
Regional tolerance compliance (CAD vs. pre-treatment): percentage of in-tolerance area across critical surface features. Figure created with BioRender.com.

Maximum surface deviation from the reference CAD model was assessed for 3D-printed coupons as a function of macro-geometry (standard vs. non-standard) and continuous CCF reinforcement. Group means confirm that non-standard geometries and CCF inclusion both elevate maximal local errors, with notable differences across conditions, as shown in Fig. 8 and supplementary Table 3. Inspection of maximum surface deviation revealed distinct error patterns associated with geometry. In non-standard coupons, localised peaks in deviation consistently originated at the ratchet-style teeth, which represent acute geometric transitions and regions of high curvature. By contrast, for standard geometry coupons, the highest deviations were not attributable to discrete features but rather to subtle, global warping and bowing of the planar surfaces. Despite this, the variation in tolerance fulfilment between the groups remained insignificant (Fig. 9).

A two-way ANOVA (Geometry × CCF) revealed significant main effects of both geometry (*p* = 0.0139) and carbon fibre inclusion (*p* = 1.14 × 10⁻⁴) on maximal surface deviation, indicating that both factors independently increase the maximum discrepancy between the printed and designed model. The interaction term was not significant (*p* = 0.272), suggesting the effects of geometry and CCF inclusion are primarily additive rather than synergistic (supplementary Table 5).

Assumption checks revealed that residuals were normally distributed (Lilliefors *p* = 1.00), but Levene’s test indicated significant heterogeneity of variances (*p* = 0.0001), violating the homoscedasticity assumption. Accordingly, results were cross-validated with a SRH rank ANOVA, which confirmed both geometry (*p* = 1.28 × 10⁻⁴) and CCF inclusion (*p* = 4.27 × 10⁻⁷) as robustly significant, and the interaction as marginal (*p* = 0.0585).

Post hoc Tukey HSD tests (supplementary Table 6) clarified these effects. Among standard geometry specimens, CCF-reinforced samples exhibited significantly greater maximum deviation than non-reinforced counterparts (mean difference = 0.367 mm, *p* = 0.00253 on raw values; rank-based difference = 19.1, *p* = 9.08 × 10⁻⁶). Similarly, among non-standard specimens, CCF-reinforced coupons deviated more than non-reinforced ones (difference = 0.466 mm, *p* = 1.20 × 10⁻⁴; ranks: 24.6, *p* = 6.04 × 10⁻⁸). Between geometries, non-standard samples with CCF deviated most, but the direct geometry-by-CCF interaction did not reach statistical significance after correction. Regional analysis of surface deviation maps demonstrated that, in non-standard coupons, maximum errors consistently localised to the ratchet-style teeth, whereas in standard coupons, maximal deviations were distributed more globally, reflecting diffuse warping of planar surfaces. Differences between standard and non-standard geometry for non-reinforced samples were marginal (raw *p* = 0.0595; ranks: *p* = 0.0005).

### Dimensional comparison between the printed models pre- and post-treatment

**Fig. 10:**
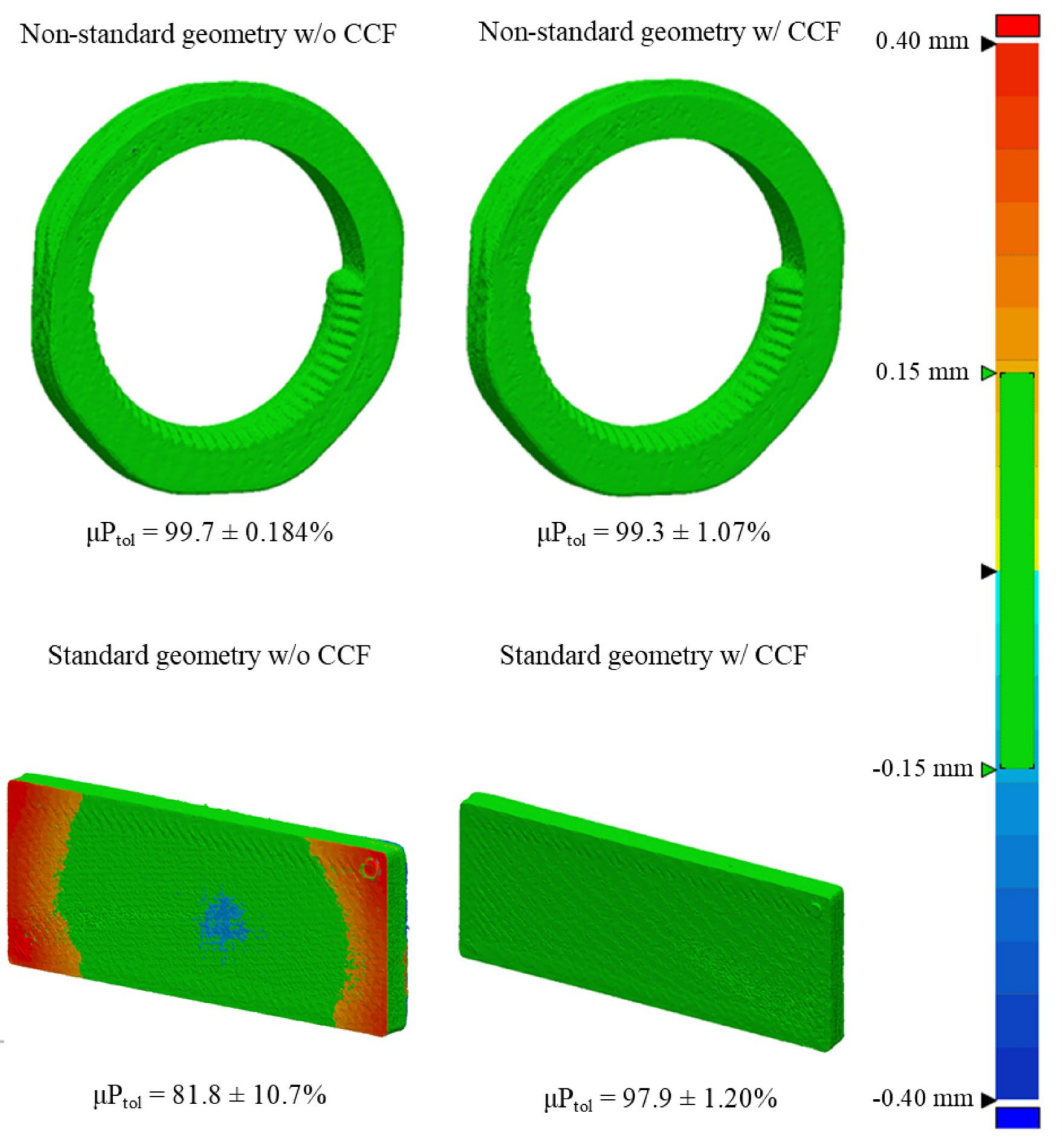
Representative surface-deviation heatmap comparing the as-printed part to the ethanol-disinfected part.

**Fig. 11:**
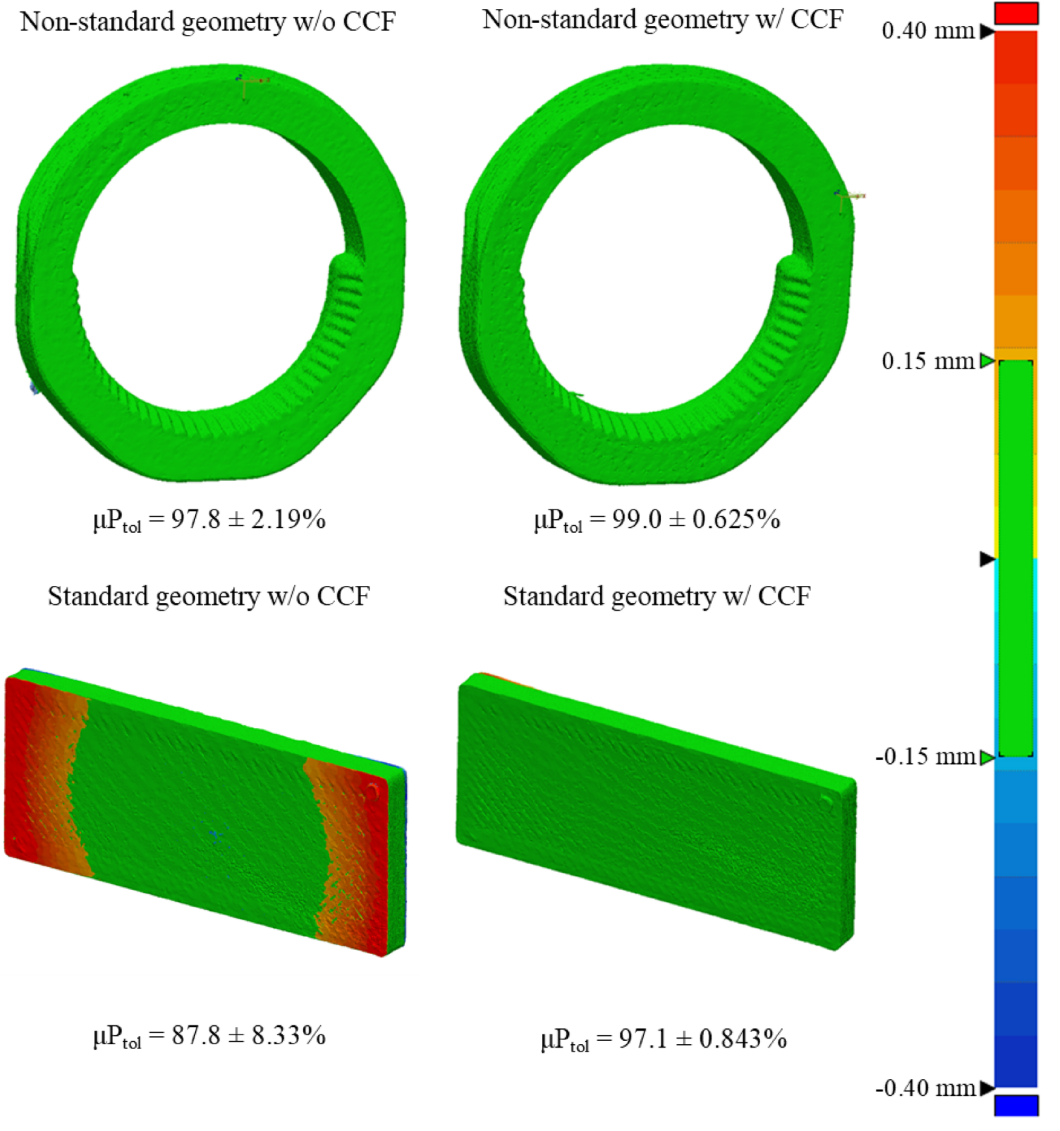
Representative surface-deviation heatmap comparing the as-printed part to the autoclave-sterilised part.

**Fig. 12:**
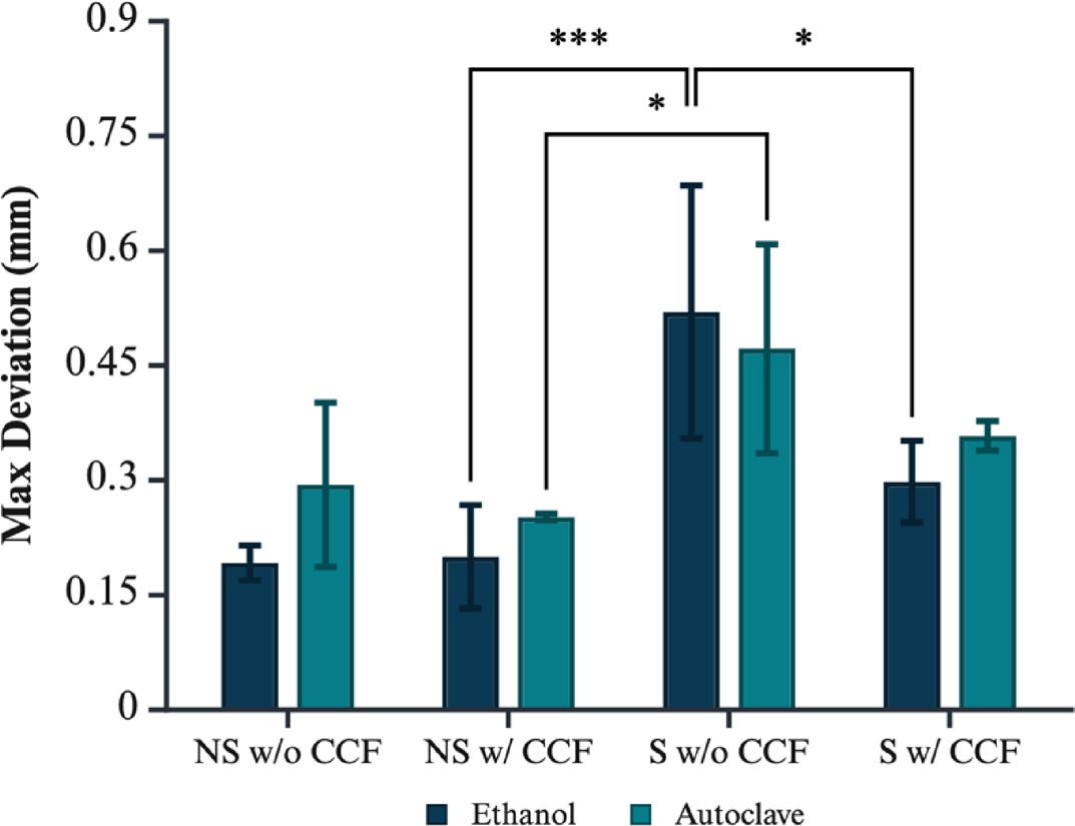
Maximum global deviation (pre- vs. post-treatment) for ethanol and autoclave cohorts, by geometry and CCF reinforcement. Significance markers: *p* < 0.05 (*), *p* < 0.01 (**), *p* < 0.001 (***). Figure created with BioRender.com.

**Fig. 13:**
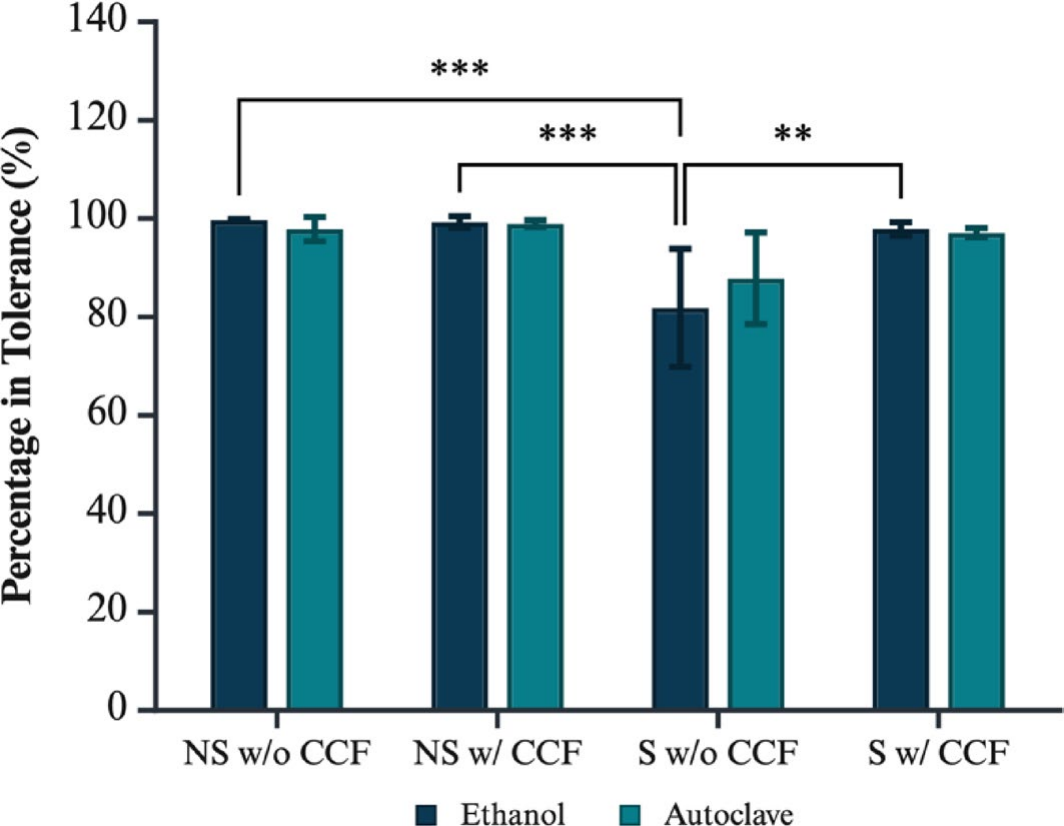
Post-treatment percentage of surface area within tolerance (pre- vs. post-ethanol/autoclave), stratified by geometry and CCF reinforcement. Significance markers: *p* < 0.01 (**), *p* < 0.001 (***). Figure created with BioRender.com.

Segmented samples from micro-CT imaging were used to assess the dimensional accuracy of printed models against each other before and after treatment, as shown in Figs. 10 and 11. Descriptive statistics are recorded in supplementary Tables 4 and the corresponding data can be visualised in Figs. 12 and 13.

A comprehensive three-way ANOVA was conducted to assess the effects of geometry (standard vs. non-standard), CCF reinforcement, and sterilisation method (autoclave vs. ethanol) on the dimensional deviation observed between the printed models and their reference geometry following treatment. Full summary statistics and post-hoc contrasts are presented in supplementary Table 5.

The analysis revealed a highly significant main effect of geometry on dimensional deviation (*p* = 6.32 × 10⁻⁷), indicating that the complexity of the printed geometry substantially influenced shape fidelity. Standard, planar plates consistently demonstrated greater dimensional error than their non-standard, curved counterparts, underscoring the greater susceptibility of simpler geometries to distortion through the print–sterilise workflow. Carbon fibre reinforcement was also a significant factor (*p* = 0.00290), with CCF-reinforced parts displaying improved dimensional stability across the cohort. In contrast, the main effect of sterilisation method was not significant (*p* = 0.160), suggesting that, on average, neither autoclaving nor ethanol disinfection introduced substantial additional deviation within the studied parameter space.

A significant interaction was observed between geometry and CCF reinforcement (*p* = 0.0132), demonstrating that the benefit of fibre reinforcement depended on the underlying geometry. Post-hoc Tukey HSD (supplementary Table 6) contrasts showed that in the standard geometry, the presence of CCF rings reduced mean deviation by 0.167 mm relative to non-reinforced plates (*p* = 0.00129). The difference was even more pronounced when comparing standard, non-reinforced plates to the non-standard, CCF-reinforced parts, with a reduction of 0.269 mm (*p* < 0.001). However, for non-standard geometries, there was no significant difference in deviation between CCF-reinforced and non-reinforced samples (*p* = 0.973), indicating that fibre reinforcement did not confer additional fidelity in more geometrically complex forms. These findings highlight that while fibre reinforcement substantially benefits dimensional stability in simple, planar designs, its effect is largely neutralised by the complexity of non-standard geometries.

Assumption checks revealed that residuals were normally distributed (Lilliefors *p* = 1.00), but homogeneity of variance was not met (Levene’s *p* = 0.0007), prompting validation with the variance-robust SRH rank-based ANOVA. The SRH analysis corroborated the primary findings, identifying significant main effects for both geometry (*p* = 7.44 × 10⁻⁹) and CCF reinforcement (*p* = 0.0161), as well as a main effect of sterilisation (*p* = 0.00820); however, the geometry × CCF interaction only approached significance (*p* = 0.0969), supporting the conclusion that geometry and fibre reinforcement are the dominant drivers of deviation. All samples maintained a tolerance of greater than 80% post treatment under both techniques.

## Discussion

AM is steadily re-wiring the medical device value chain, collapsing design-to-patient lead times, shortening logistic routes for critical spares, and driving cost deflation across disposables and capital equipment alike^24^. By prioritising additive methods over traditional subtractive techniques, manufacturers can develop complex, optimised components with minimal material wastage, applicable across a wide spectrum of devices, ranging from Class I to Class III certification and is projected to reach a market value of $15.35 billion by 2032^25^. Crucially, the digital genesis of AM parts dovetails with three computational paradigms that are rapidly permeating biomedical engineering^26^: biomimetic design (reverse-engineering nature’s load paths)^27^; field-driven design (coupling anatomy-specific voxel fields to localised stiffness or porosity)^28^; and generative design (AI-driven heuristic searches for lightweight topologies)^29^. Collectively, these methodologies not only enhance the functionality and integration of medical devices but also herald a new era in healthcare engineering, mirroring their recent transformative impact in the automotive and aerospace industries.

Within this broader movement, the medical sector’s appetite for strength-to-weight ratioing—so-called lightweighting—of medical components places a premium on polymers that can challenge metal incumbents^30–34^. AM facilitates the utilisation of innovative materials that are challenging to process through traditional means. Thermoplastics such as polyphenylsulfone (PPSU), nylon, polyetheretherketone (PEEK), their composite counterparts, and CFRPs like carbon, glass, and Kevlar^®^ are particularly well-suited for FFF, providing the strength and lightweight properties ideal for medical applications.

Despite these advancements, significant challenges persist in integrating additively manufactured polymeric devices into mainstream medical instrumentation. Issues such as sterilisation and disinfection, ensuring appropriate mechanical properties, and meeting stringent quality assurance (QA) and QC requirements present substantial obstacles^35–37^. To address these challenges, composite polymers with reinforcement capabilities are being explored for their improved strength-to-weight ratio, high resistance to chemicals, sterilisability, radiolucency, and acceptable dimensional resolution^2,5–7^. Additionally, compliance with critical certifications – such as ISO 14155 for clinical trial regulations, ISO 13485 for quality management systems, ISO 10993 for biocompatibility, and ISO 14971 for risk management – is often recommended or required to ensure market readiness.

A significant hurdle in advancing a medical device from development to market is attaining ISO 13485 certification. Quality management systems (QMS) in the context of AM for medical devices implement rigorous processes to ensure repeatability and geometric accuracy. These processes include precise alignment and geometric comparison of printed parts to their CAD models, as well as comprehensive evaluations of mechanical properties and the effects of sterilisation and disinfection. Fabrication accuracy, which measures the congruence between the printed samples and their corresponding CAD models, is a fundamental aspect of QA/QC. Thus, this study aimed to evaluate the impact of steam-autoclave sterilisation and ethanol disinfection on the dimensional accuracy of 3D-printed parts made from carbon fibre nylon composites, with and without CCF reinforcement.

Our findings indicate that neither sterilisation nor disinfection had a significant effect on the dimensional accuracy of the printed carbon fibre nylon parts. Pre-treatment analysis, compared against the CAD model, revealed minimal void space – well below the described 4% void identified by Saeed et al.^38^ – for parts with and without CCF. Dimensional accuracy was relatively consistent across all parts. However, regional surface deviation analysis highlighted significant differences between macro-geometric features. Specifically, non-standard geometries consistently exhibited the largest deviations around ratchet-style teeth features, exceeding other regions by up to 0.47 mm when reinforced with CCF (*p* < 0.001). By contrast, the standard geometry predominantly displayed global warping as the primary mode of deformation, with maximum deviations typically observed at peripheral edges rather than in detailed internal features. This localised pattern indicates that geometric complexity and fibre reinforcement distinctly influence where deviations concentrate, affecting final accuracy significantly. The absence of significant interaction effects between geometry and fibre reinforcement (interaction term *p* = 0.27) indicates that these factors independently increase surface deviation, suggesting additive rather than combined effects. Thus, addressing each factor separately through targeted design and reinforcement strategies will likely yield the most effective improvements in dimensional accuracy. There are further limitations inherent in the printing process and the resolution of the print nozzle, which had a diameter of 0.40 mm. The nozzle diameter restricts the maximum turning radius of the extruded filament, highlighting the need for manufacturers to consider printing resolution when designing parts that do not require post-processing to enhance dimensional accuracy. Interestingly, the threaded features did not exhibit significant dimensional differences despite the constraints posed by a layer height of 0.15 mm. While threads in 3D printing can achieve reasonable accuracy, their precision is influenced by factors such as nozzle size and layer height, which impact resolution and surface finish.

The analysis of parts before and after treatment revealed differences in void space and dimensional accuracy. Voids predominantly arise from air entrapment and moisture absorption during material storage and processing. Post-treatment, both techniques showed that the parts experienced a minimal reduction in void space – less than 1% – except for the non-standard geometry without CCF during autoclave sterilisation, where the void space marginally increased on average. The localisation of void formation also varied substantially with geometry and reinforcement. Non-standard geometries printed without fibre reinforcement uniquely displayed significantly lower void fractions (~ 0.95% points lower, *p* < 0.001), suggesting enhanced consolidation in the complex areas due to more uniform cooling and fewer internal stresses. Conversely, fibre-reinforced specimens, particularly with standard geometry, displayed consistently higher void contents, possibly attributable to air entrapment around fibres. Similarly, the surface deviation of the parts after treatment for both techniques exhibited minor differences. Autoclave sterilisation generally induced greater warping compared to ethanol disinfection, except for the standardised geometry without CCF, which demonstrated similar behaviour across both methods. Parts reinforced with CCF exhibited less deviation in both treatment methods; however, CCF did not significantly reduce deformation during autoclaving. The tensile and compressive thermal stresses experienced by parts during autoclaving are likely exacerbated by the anisotropic nature of 3D-printed layers and the material properties of nylon and carbon fibre^39^. While CCF is effective in enhancing tensile strength, it may have a limited impact on reducing thermal deformation during autoclaving due to its lower coefficient of thermal expansion compared to the nylon composite polymer matrix and its anisotropic thermal conductivity^39^. As such, treatment methods did not significantly affect the void fraction, indicating that the changes in void fraction were primarily driven by the geometry and material composition of the samples rather than the treatment type used. The substantial standard deviations observed in specific conditions (e.g., standard geometry with CCF and non-standard geometry without CCF) primarily originated from geometrically complex features such as ratchet-style teeth. Variability was exacerbated by inherent fluctuations in layer deposition consistency, thermal gradients between successive prints, and anisotropic cooling stresses, all of which became more pronounced in regions of abrupt geometric transitions. These effects manifest more prominently in areas with abrupt geometric transitions, exacerbating variability in accuracy and void presence between consecutive prints.

The inclusion of CCF, regardless of geometry, tended to increase the void fraction, meaning that that both geometry and CCF inclusion significantly influence the void fraction of 3D-printed parts. Therefore, the choice to incorporate certain design elements and specific printing parameters can significantly influence the dimensional accuracy of additively manufactured parts both before and after treatment. Smaller layer heights enhance the dimensional accuracy of printed parts but may increase the likelihood of warping due to more thermal cycles – a consequence of additional layers – leading to greater residual stress from the polymer’s heating and cooling processes^40,41^. The introduction of CFRPs may help alleviate these issues by increasing the tensile strength of parts during printing^39^. Other techniques, such as adding rafts and brims, may also help reduce warping by increasing the anchoring support of the part to the build plate; however, this comes at the expense of increased post-processing and material wastage. Balancing high resolution with structural integrity is essential for optimal part performance.

Additionally, the heat deflection temperature of materials, such as carbon fibre nylon composite, is crucial in determining their performance during steam sterilisation. Onyx^®^ softens at around 145 °C, and exposure to the autoclave temperature of 121 °C for an extended duration approaches this limit, leading to the tempering and partial deformation of parts^42,43^. Another high-performance polymer, PEEK, has a glass transition temperature of 145 °C and melting temperature of 343 °C. Similarly, in a study from Sharma et al.^12^they found no discernible difference in dimensional characteristics after undergoing steam sterilisation at 134 °C for 30–40 min. Conversely, low-temperature thermoplastics such as PLA, ABS, HIPS, and PETG exhibit significant deformation or melting during heat-based sterilisation methods, rendering them unsuitable for such applications^13–15^. The sterilisability of SLA-printed resins varies significantly across studies. While many report deformation and cracking after autoclaving^13^others observe negligible dimensional changes depending on the resin’s composition and its resistance to heat^11,14,16^. Thus, the outcome of autoclave sterilisation for photopolymer resins is highly dependent on the specific resin formulation and its thermal properties^9^.

Although thermal exposure compromises the dimensional stability of parts, design choices and filament laydown patterns substantially influence the extent of deformation. Non-standard geometries with a concentric design exhibited better resistance to deformation compared to standardised geometries with a rectilinear design. By reducing stress concentrators, a common example being sharp corners, and opting for more natural shapes, thermomechanical stress is dispersed easier, leading to less structural deformation. The longer, more distributed laydown of the filament in parts, as seen in the standardised design, causes edges to warp as it shrinks due to reduced structural stability and inhomogeneous cooling. Additionally, denser infill patterns can exacerbate internal stress accumulation, causing parts to warp as they cool unevenly^44^. In contrast, concentric designs distribute thermal stresses more evenly during heating and cooling, minimising deformation. Standardised samples with greater axial surface area to height ratios are particularly prone to warping due to reduced peripheral support, further highlighting the importance of geometric considerations^40,45^.

Chemical disinfection methods, such as ethanol treatment, interact differently with polymeric materials, with their effects highly dependent on the material composition and its response to the disinfectant^46–48^. Material compounds in filaments are known to react to chemical agents such as 70% ethanol. For example, nylon parts swell when exposed to 70% ethanol due to water molecule infiltration into amorphous regions, increasing polymer chain mobility and potentially causing permanent deformation if the nylon relaxes into a new shape during drying^42^. This effect was observed in standardised geometries without CCF, underscoring the role of design in deformation behaviour during disinfection. Conversely, CCF-reinforced geometries exhibited reduced deformation, likely due to the increased tensile stiffness imparted by the carbon fibre reinforcement. Told et al.^13^ reported that ethanol disinfection caused HIPS to become more brittle and sturdy without inducing swelling or other visible deformations. In contrast, PLA exhibited significant brittleness, fragility, and cross-sectional swelling due to its hygroscopic nature.

Beyond ethanol and autoclaving, other sterilisation techniques – including ethylene oxide (EtO), sporicidal chemicals, gamma irradiation, and electron beam (E-Beam) sterilisation – offer alternative solutions. EtO is particularly effective for sterilising common 3D-printed polymers such as PLA, PETG, PP, nylon, and various resins, owing to its low operating temperatures and broad material compatibility^15^. Despite its suitability for polymeric medical devices, especially those incorporating electronic components, EtO has significant drawbacks. Its lengthy cycle time, high cost, and potential risks to both patients and staff make it less appealing compared to faster, more cost-effective methods when applicable^15^. Similarly, plasma sterilisation and gamma irradiation have demonstrated efficacy with many polymeric materials^14,15^. However, gamma irradiation is not widely available, limiting its practical use^13,15^. Furthermore, radiation from plasma, gamma, and E-Beam sterilisation can degrade semiconductors, rendering these methods unsuitable for medical devices with integrated electronic components^49^.

This study has several limitations that highlight opportunities for future research. Firstly, the investigation exclusively focused on ethanol disinfection and autoclave sterilisation, thus excluding other relevant sterilisation techniques such as EtO, gamma irradiation, and electron beam sterilisation. Evaluating these additional sterilisation methods would enhance the applicability of results across diverse clinical scenarios. Secondly, the material scope was restricted to carbon fibre-reinforced nylon composites; extending the analysis to include other high-performance composite materials, such as glass or Kevlar^®^ fibres, could reveal alternative material strategies suited for specific chemical, mechanical, or thermal requirements. Thirdly, the geometric diversity studied was limited to two specific designs, which do not fully capture the intricate complexity commonly encountered in medical device manufacturing. Future research should therefore incorporate a broader array of intricate geometries, enabling a deeper understanding of how detailed design features influence deformation and void formation under sterilisation conditions. Additionally, performing detailed regional analyses across these varied geometries would facilitate precise mapping of deformation hotspots and localised void concentrations. Future work should also quantify non-mechanical surface and interface characteristics as part of routine QC to complement the geometric and mechanical metrics reported here. Finally, assessing variability and repeatability across multiple prints would help quantify the extent of dimensional and structural inconsistencies arising from printer-specific thermal fluctuations and mechanical variability.

## Conclusions

The integration of AM in medical device production offers significant potential for innovation, particularly with advanced polymers and bio-inspired designs. This study demonstrates the importance of optimising sterilisation and disinfection processes to preserve dimensional accuracy and structural integrity of AM-produced parts. Despite insignificant effects of treatment on all parts assessed in this study, CFR in carbon-fibre-nylon composites shows additional capability in minimising the slight deformation caused by sterilisation and disinfection methods, particularly with ethanol washing. However, autoclave sterilisation can induce warping, necessitating careful material selection and design optimisation. Ultimately, the inclusion of CCF and design optimisation were the significant factors that affected the dimensional accuracy of the carbon fibre nylon composite parts. Advanced sterilisation techniques, such as EtO and gamma irradiation, present alternatives for sensitive applications but face limitations in cost, availability, and compatibility with electronic components. Future efforts should focus on developing sterilisation-resistant materials, refining design strategies to mitigate thermal stresses, and exploring novel sterilisation methods. By aligning materials, design, and manufacturing processes, AM can deliver reliable, sterilisable, and cost-effective medical devices, paving the way for broader adoption in healthcare.

## Supplementary Information

Below is the link to the electronic supplementary material.

Supplementary Material 1

## Data Availability

The datasets generated during and/or analysed during the current study are available from the corresponding author on reasonable request.
