# Supplementary material for: Influence of geometry, reinforcement, and sterilisation on the dimensional accuracy of additively manufactured carbon fibre-reinforced nylon composites
Source: Sci Rep. 2025 Oct 1;15:34268. doi: 10.1038/s41598-025-16696-w (PMC12489055; doi:10.1038/s41598-025-16696-w)
Supplement: Supplementary file 1 — Supplementary Material 1 [file 41598_2025_16696_MOESM1_ESM.pdf]

Supplementary Table 1. Descriptive statistics for the in-silico assessment of void formation within 3D-printed carbon fibre reinforced nylon composite parts. NS w/ CCF, non-standard geometry with continuous carbon fibre; S w/CCF, standard geometry with continuous carbon fibre; NS w/o CCF, non-standard geometry without continuous carbon fibre; S w/o CCF, standard geometry without continuous carbon fibre.

	**NS w/ CCFF**		**S w/ CCFF**		**NS w/o CCFF**		**S w/o CCFF**	
**Treatment**	**Pre-**	**Post-**	**Pre-**	**Post-**	**Pre-**	**Post-**	**Pre-**	**Post-**
Ethanol	3.1965	2.9652	3.3081	3.3166	2.7182	1.5662	3.3629	2.7420
	3.0230	2.9245	3.0245	2.9174	1.7797	1.8075	3.3797	3.2467
	2.8877	2.8049	2.9672	2.9987	2.4401	2.4321	3.0490	2.7228
	2.9441	2.8395	2.1839	1.9046	1.5239	1.4009	3.1136	3.1248
	3.1727	3.2867	3.6959	2.4689	3.0559	1.5889	3.7569	3.0365
**Average**	**3.0448**	**2.9642**	**3.0359**	**2.7212**	**2.3036**	**1.7591**	**3.3324**	**2.9745**
**Standard Deviation**	**0.1222**	**0.1712**	**0.4981**	**0.4900**	**0.5725**	**0.3605**	**0.2496**	**0.2088**
Autoclave	3.1910	3.1563	3.6793	3.4145	1.8011	2.5592	3.1382	2.8748
	3.0277	2.7808	3.3629	3.2817	2.6328	2.3074	3.3812	2.8709
	3.0008	2.9015	2.8628	2.9153	1.7383	1.5260	3.0700	2.9309
	2.8374	2.7907	2.1969	2.0098	2.6017	2.2234	3.1354	2.8673
	3.3843	3.1462	3.4673	2.4304	1.8982	2.7087	3.5432	3.2580
**Average**	**3.0882**	**2.9551**	**3.1138**	**2.8103**	**2.1344**	**2.2649**	**3.2536**	**2.9604**
**Standard Deviation**	**0.1857**	**0.1657**	**0.5311**	**0.5260**	**0.3976**	**0.4082**	**0.1795**	**0.1506**

Supplementary Table 2. Descriptive statistics of the geometric dimensional comparison between CAD models and 3D-printed models comprised of carbon fibre nylon composite both with and without the inclusion of CCF. This comparison assesses the maximum geometrical deviation as well as the percentage of which the printed model is within a 0.15 mm tolerance of the original CAD model

	**Max Surface Deviation (mm)**		**Percentage within 0.15 mm Tolerance (%)**	
	**Average**	**Standard Deviation**	**Average**	**Standard Deviation**
**NS w/o CCF**	2.2605	0.0157	80.4806	3.7868
**NS w/ CCF**	2.2677	0.0081	75.4763	1.1928
**S w/o CCF**	2.1684	0.0013	77.7261	4.6743
**S w/CCF**	1.8893	0.2019	81.3274	3.8850

Supplementary Table 3. Descriptive statistics of the surface comparison between CAD models and 3D-printed models comprised of carbon fibre nylon composite both with and without the inclusion of CCF. This assesses the maximum surface deviation as well as the percentage of which the printed model is within a 0.15 mm tolerance of the original CAD model

	**Max Surface Deviation (mm)**		**Percentage within 0.15 mm Tolerance (%)**	
	**Average**	**Standard Deviation**	**Average**	**Standard Deviation**
**NS w/o CCF**	0.5638	0.2232	93.8443	2.1802
**NS w/ CCF**	0.7805	0.2316	93.0649	2.5074
**S w/o CCF**	0.3142	0.0252	93.4572	2.0683
**S w/CCF**	0.6813	0.2444	88.3950	9.5867

Supplementary Table 4. Descriptive statistics of the dimensional comparison between 3D-printed models comprised of carbon fibre nylon composite both with and without the inclusion of CCF before and after treatment through ethanol disinfection or autoclave sterilisation. Dimensional comparison assesses the maximum surface deviation as well as the percentage of which the treated model is within a 0.15 mm tolerance of the original 3D-printed model

	**Treatment**	**Max Surface Deviation (mm)**		**Percentage within 0.15 mm Tolerance (%)**	
		**Average**	**Standard Deviation**	**Average**	**Standard Deviation**
**NS w/o CCF**	**Ethanol**	0.1932	0.0217	99.6704	0.1843
	**Autoclave**	0.2922	0.0960	97.8377	2.1925
**NS w/ CCF**	**Ethanol**	0.1992	0.0602	99.3061	1.0708
	**Autoclave**	0.2516	0.0054	98.9793	0.6250
**S w/o CCF**	**Ethanol**	0.5175	0.1476	81.8380	10.7397
	**Autoclave**	0.4705	0.1197	87.8289	8.3277
**S w/ CCF**	**Ethanol**	0.2971	0.0478	97.9105	1.2006
	**Autoclave**	0.3570	0.0183	97.1175	0.8432

Supplementary Table 5. Summary of statistical analyses for void fraction, dimensional accuracy, and surface deviation

	Analysis	*Factor / Interaction*	*F / H*	*p-value*	*Significance*	*Assumption Tests*	*Key Post Hoc Contrasts[Diff, 95% CI], p*	*Interpretation*	*Summary*
Void Fraction	Three-way ANOVA	Geometry	F = 9.90	0.00360	**Yes**	Lilliefors p = 0.000 (reject)<br><br>Levene p = 0.0524 (assume)	Standard+CCF vs Non-Standard–CCF: 0.754 [0.290, 1.217], p = 0.000611	Geometry increases void fraction	The use of a standard geometry elevates void formation
		Continuous Carbon Fibre (CCF)	F = 9.52	0.00420	**Yes**		Non-Standard+CCF vs Non-Standard–CCF: 0.948 [0.485, 1.412], p = 2.35e-05	CCF increases void fraction, strong effect	CCF addition increases porosity
		Sterilization	F = 1.39	0.247	No		—	Sterilization not significant	No effect of sterilization on porosity
		Geometry × CCF	F = 22.6	4.07e-05	**Yes**		—	Strong synergy between geometry and CCF	CCF effect depends on geometry
		Geometry × Sterilization	F = 0.757	0.391	No		—	No interaction	No evidence of geometry–sterilization interaction
		CCF × Sterilization	F = 0.727	0.400	No		—	No interaction	No evidence of CCF–sterilization interaction
		Geometry × CCF × Steril	F = 1.64	0.210	No		—	No three-way interaction	No combined effect of all three factors

	Scheirer–Ray–Hare	Geometry (H)	H = 7.51	0.00990	**Yes**	—	Standard+CCF vs Non-Standard–CCF: Δrank = 16.2 [4.88, 27.5], p = 0.00266	Non-parametric test confirms geometry effect	Geometry main effect robust to assumption violations
		CCF (H)	H = 7.51	0.00990	**Yes**		Non-Standard+CCF vs Non-Standard–CCF: Δrank = 18.9 [7.58, 30.2], p = 0.000440	Non-parametric test confirms CCF effect	CCF main effect robust to assumption violations
		Sterilization (H)	H = 0.139	0.712	No		—	—	Sterilization effect not robust (remains non-significant)
		Geometry × CCF (H)	H = 13.4	9.14e-04	**Yes**		—	Synergistic interaction confirmed	Geometry and CCF interact to modulate porosity
		Geometry × Steril (H)	H = 0.0183	0.893	No		—	—	No interaction between geometry and sterilization
		CCF × Steril (H)	H = 0.165	0.687	No		—	—	No interaction between CCF and sterilization
		Three-way (H)	H = 0.331	0.569	No		—	—	No three-way interaction
Pre-Post Deviation	Three-way ANOVA	Geometry	F = 38.3	6.32e-07	**Yes**	Lilliefors p = 1.00 (reject)	Standard+CCF vs Non-Standard–CCF: 0.955	Significant geometric	Geometry is the largest determinant

					Levene p = 0.0007 (reject)	[0.492, 1.419], p = 2.08e-05	effect on deviation	of post-treatment error
	CCF	F = 10.4	0.00290	**Yes**		—	Significant CCF effect	Carbon fibre increases deformation
	Sterilization	F = 2.07	0.160	No		—	Not significant	Sterilization has no clear impact
	Geometry × CCF	F = 6.88	0.0132	**Yes**		—	Interaction present	Effect of CCF depends on geometry
	Geometry × Sterilization	F = 1.47	0.234	No		—	—	No interaction between geometry and sterilization
	CCF × Sterilization	F = 0.279	0.601	No		—	—	No interaction between CCF and sterilization
	Geometry × CCF × Steril	F = 1.81	0.188	No		—	—	No three-way interaction
Scheirer–Ray–Hare	Geometry (H)	H = 60.3	7.44e-09	**Yes**	—	Standard+CCF vs Non-Standard–CCF: Δrank = 20.5 [12.2, 28.8], p = 8.49e-07	Non-parametric confirmation	Geometry main effect is robust
	CCF (H)	H = 6.46	0.0161	**Yes**		—	—	CCF effect robust to assumption violations

		Sterilization (H)	H = 7.95	0.00820	**Yes**		—	Noted in non-parametric only	Weak effect of sterilization on ranks only
		Geometry × CCF (H)	H = 2.92	0.0969	No		—	—	No interaction on ranks
		Geometry × Steril (H)	H = 2.92	0.0969	No		—	—	—
		CCF × Steril (H)	H = 0.077	0.783	No		—	—	—
		Three-way (H)	H = 1.23	0.276	No		—	—	—
CAD–Printed Deviation	Two-way ANOVA	Geometry	F = 48.5	3.63e-08	**Yes**	Lilliefors p = 1.00 (cannot reject)<br><br>Levene p = 0.0000 (reject)	Standard+CCF vs Non-Standard–CCF: 0.371 [0.243, 0.500], p = 1.93e-08	Geometry and CCF affect CAD-printed deviation	Non-standard geometry increases deviation
		CCF	F = 16.2	2.80e-04	**Yes**		—	—	Carbon fibre increases deviation
		Geometry × CCF	F = 17.98	1.49e-04	**Yes**		—	—	Interaction: CCF and geometry modulate deviation
	Scheirer–Ray–Hare	Geometry (H)	H = 191	5.66e-16	**Yes**	—	Standard+CCF vs Non-Standard–CCF: Δrank = 23.1 [17.5, 28.6], p = 1.37e-12	Main effect robust	—
		CCF (H)	H = 4.45	0.0420	**Yes**		Standard+CCF vs Standard–CCF:	—	—

							Δrank = 10.0 [4.49, 15.5], p = 1.20e-04		
		Geometry × CCF (H)	H = 23.1	2.72e-05	**Yes**		—	—	—
Max Surface Deviation	Two-way ANOVA	Geometry	F = 6.69	0.0139	**Yes**	Lilliefors p = 1.00 (cannot reject)<br><br>Levene p = 0.0001 (reject)	Standard+CCF vs Standard–CCF: 0.367 [0.110, 0.624], p = 0.00253	Geometry and CCF both affect max surface deviation	Non-standard geometry increases peak surface deviation
		CCF	F = 18.7	1.14e-04	**Yes**		Non-Standard+CCF vs Standard–CCF: 0.466 [0.209, 0.723], p = 1.20e-04	—	Carbon fibre amplifies max surface deviation
		Geometry × CCF	F = 1.24	0.272	No		—	No significant interaction	No synergy on max deviation
	Scheirer–Ray–Hare	Geometry (H)	H = 18.4	1.28e-04	**Yes**	—	Standard+CCF vs Standard–CCF: Δrank = 19.1 [10.1, 28.1], p = 9.08e-06	Main effect robust	Geometry effect confirmed with non-parametric analysis
		CCF (H)	H = 37.9	4.27e-07	**Yes**		Non-Standard+CCF vs Standard–CCF: Δrank = 24.6 [15.6, 33.6], p = 6.04e-08	—	CCF effect confirmed with non-parametric analysis

	Geometry × CCF (H)	H = 3.82	0.0585	No	—	—	No significant interaction

Supplementary Table 6. Summary of post-hoc pairwise comparisons for void fraction, dimensional accuracy, and surface deviation; significance markers: $p < 0.05$ (*), $p < 0.01$ (**), $p < 0.001$ (***), ns = non-significant.

Outcome	***Group A***	***Group B***	***Mean Diff.***	***95% CI (Lower → Upper)***	***p-value***	***Sig.***
Void fraction (% points)	Standard + CCF	Non-std + CCF	–0.1943	–0.6578 → 0.2691	0.6704	n.s.
	Standard + CCF	Standard – CCF	–0.2017	–0.6652 → 0.2618	0.6442	n.s.
	Standard + CCF	Non-std – CCF	0.7538	0.2903 → 1.2172	0.000611	***
	Non-std + CCF	Standard – CCF	–0.0073	–0.4708 → 0.4561	0.99997	n.s.
	Non-std + CCF	Non-std – CCF	0.9481	0.4846 → 1.4116	2.35×10^{-5}	***
	Standard – CCF	Non-std – CCF	0.9554	0.4920 → 1.4189	2.08×10^{-5}	***
Pre–Post deviation (mm)	Standard + CCF	Non-std + CCF	0.1016	–0.0076 → 0.2109	0.0758	n.s.
	Standard + CCF	Standard – CCF	–0.1670	–0.2763 → –0.0577	0.00129	**
	Standard + CCF	Non-std – CCF	0.0843	–0.0250 → 0.1936	0.1780	n.s.
	Non-std + CCF	Standard – CCF	–0.2686	–0.3779 → –0.1593	9.50×10^{-7}	***
	Non-std + CCF	Non-std – CCF	–0.0173	–0.1266 → 0.0919	0.9730	n.s.
	Standard – CCF	Non-std – CCF	0.2513	0.1420 → 0.3606	3.24×10^{-6}	***
CAD → Pre deviation (mm)	Standard + CCF	Non-std + CCF	–0.3785	–0.5071 → –0.2498	1.24×10^{-8}	***
	Standard + CCF	Standard – CCF	–0.2791	–0.4078 → –0.1505	6.51×10^{-6}	***
	Standard + CCF	Non-std – CCF	–0.3712	–0.4999 → –0.2426	1.93×10^{-8}	***
	Non-std + CCF	Standard – CCF	0.0993	–0.0293 → 0.2279	0.1792	n.s.
	Non-std + CCF	Non-std – CCF	0.0072	–0.1214 → 0.1359	0.9987	n.s.
	Standard – CCF	Non-std – CCF	–0.0921	–0.2207 → 0.0366	0.2346	n.s.
Max surface deviation (mm)	Standard + CCF	Non-std + CCF	–0.0992	–0.3562 → 0.1577	0.7272	n.s.
	Standard + CCF	Standard – CCF	0.3671	0.1102 → 0.6241	0.00253	**
	Standard + CCF	Non-std – CCF	0.1175	–0.1394 → 0.3745	0.61095	n.s.

Non-std + CCF	Standard – CCF	0.4664	0.2095 → 0.7233	1.20×10^{-4}	***
Non-std + CCF	Non-std – CCF	0.2168	–0.0401 → 0.4737	0.1236	n.s.
Standard – CCF	Non-std – CCF	–0.2496	–0.5065 → 0.0073	0.0595	n.s.
